# Effectiveness and Safety of Guselkumab in Patients With Moderate‐to‐Severe Plaque Psoriasis in Real‐World Practice in Korea: A Prospective, Multicenter, Observational, Postmarketing Surveillance Study

**DOI:** 10.1111/1346-8138.17757

**Published:** 2025-04-28

**Authors:** Bong Seok Shin, Miri Kim, Moo Kyu Suh, Young Bok Lee, Sang Woong Youn, Ji Yeoun Lee, Chul Woo Kim, Ga‐Young Lee, Sang Wook Son, Kwang Ho Kim, Jihye An, Youngdoe Kim, Kwang Joong Kim, Dong Hyun Kim

**Affiliations:** ^1^ Department of Dermatology Chosun University College of Medicine Gwangju Republic of Korea; ^2^ Department of Dermatology Yeouido St. Mary's Hospital, College of Medicine, The Catholic University of Korea Seoul Republic of Korea; ^3^ Department of Dermatology Dongguk University College of Medicine Gyeongju Republic of Korea; ^4^ Department of Dermatology Uijeongbu St. Mary's Hospital, College of Medicine, The Catholic University of Korea Uijeongbu Republic of Korea; ^5^ Department of Dermatology Seoul National University College of Medicine, Seoul National University Bundang Hospital Seongnam Republic of Korea; ^6^ Department of Dermatology, College of Medicine Chungbuk National University Cheongju Chungbuk Republic of Korea; ^7^ Department of Dermatology Kangdong Sacred Heart Hospital, Hallym University College of Medicine Seoul Republic of Korea; ^8^ Department of Dermatology Kangbuk Samsung Hospital, Sungkyunkwan University School of Medicine Seoul Republic of Korea; ^9^ Department of Dermatology Korea University Ansan Hospital Ansan Republic of Korea; ^10^ Department of Dermatology Hallym University Sacred Heart Hospital, Hallym University College of Medicine Anyang Republic of Korea; ^11^ Medical Affairs, Janssen Korea Ltd. Seoul Republic of Korea; ^12^ Department of Dermatology CHA Bundang Medical Center, CHA University School of Medicine Seongnam Republic of Korea

**Keywords:** Korea, observational study, psoriasis, safety, treatment effectiveness

## Abstract

Clinical trials have demonstrated the efficacy and safety of guselkumab in patients with moderate‐to‐severe plaque psoriasis. Real‐world evidence for guselkumab in Korea is needed to establish drug safety and effectiveness under real‐world practice in this population. This study assessed the effectiveness, safety, and drug survival of guselkumab in Korean patients with moderate‐to‐severe plaque psoriasis in a real‐world clinical setting. In this prospective, non‐interventional observational study conducted at 44 clinical centers in South Korea, adult patients with moderate‐to‐severe plaque psoriasis who would receive guselkumab per the product label were enrolled. Disease assessment was performed at visits 1 (baseline, week 0), 2 (approximately week 4), and 3–7 (approximately every 8 weeks after week 4, weeks 12–44). Between February 25, 2019 and March 25, 2022, 707 patients were enrolled and 562 (79.5%) had completed the 56‐week observation period. The proportions of patients with an absolute Psoriasis Area and Severity Index (PASI) score of ≤ 3, ≤ 2, or ≤ 1 reached maximums of 96.0%, 86.7%, and 59.0%, respectively, at visit 7. A significantly greater proportion of biologic‐naïve (vs. biologic‐experienced) patients achieved absolute PASI ≤ 3 by visits 5–7 (*p* = 0.001 at visit 7) and absolute PASI ≤ 2 by visits 6–7 (*p* = 0.0014 at visit 7). Dermatology Life Quality Index (DLQI) scores decreased over time, with 64.1% of patients achieving DLQI 0/1 by visit 7; results were similar regardless of prior biologic therapy. The estimated drug‐survival rate at 1 year was 92.7%. Adverse events (AEs) occurred in 22.5% of patients, with an incidence rate of 42.1 per 100 patient years (PY); 14 serious AEs occurred in 13 (1.8%) patients, with an incidence rate (95% confidence interval) of 2.4 (1.2–3.7) per 100 PY. Guselkumab administered under approved label conditions was effective and well‐tolerated in Korean patients with moderate‐to‐severe plaque psoriasis in a real‐world clinical setting.

## 1 Introduction

Psoriasis is an immune‐mediated, chronic inflammatory disease that affects an estimated 125 million people, with a prevalence of 0.33%–0.6% globally [1]. In 2015, there were 233, 909 people in Korea with psoriasis, with an age‐ and sex‐standardized prevalence of 453 per 100 000; 23% of patients had moderate‐to‐severe disease [2]. The most common subtype is plaque psoriasis, characterized by sharply demarcated, erythematous, scaly plaques, which accounts for ~80%–90% of patients [2, 3]. Psoriasis is associated with a wide spectrum of comorbid conditions that are not limited to skin manifestations, such as psoriatic arthritis, cardiovascular diseases, metabolic diseases, gastrointestinal diseases, chronic kidney diseases, and mental health diseases, thus affecting many aspects of patients' lives and severely impacting overall quality of life [1].

Standard treatments for moderate‐to‐severe psoriasis, defined by 3% to > 10% of body surface area (BSA) involvement, include phototherapy, and systemic non‐biologic and biologic therapies [4, 5, 6]. Among these, biologic therapy has demonstrated a favorable benefit‐to‐risk ratio and represents a significant advancement in the treatment of psoriasis [6]. Currently, there are four classes of biologics, which include tumor necrosis factor‐α inhibitors, interleukin (IL)‐12/IL‐23 inhibitors, IL‐17 inhibitors, and IL‐23 inhibitors [6].

Guselkumab is a fully human monoclonal antibody that targets the p19 subunit of IL‐23 and inhibits IL‐23‐mediated signaling. Guselkumab has been approved in several countries to treat patients with moderate‐to‐severe plaque psoriasis who are candidates for systemic therapy or phototherapy and patients with active psoriatic arthritis, with an additional indication in Japan and Korea for the treatment of palmoplantar pustulosis [7, 8, 9, 10]. The efficacy and well‐tolerated safety profile of guselkumab in patients with moderate‐to‐severe psoriasis have been demonstrated by evidence from phase III randomized controlled clinical trials [11, 12, 13, 14, 15, 16]. Guselkumab has also shown superior efficacy over adalimumab and placebo; the response to guselkumab was durable and the safety profile remained well‐tolerated over long‐term treatment up to week 48 in pivotal studies in psoriasis [11, 12]. Additionally, guselkumab maintenance therapy was effective in patients who did not respond to adalimumab [11, 12]. Clinical response to guselkumab was sustained with open‐label extension maintenance therapy for up to 5 years; consistent results were observed in the Asian subpopulation [13, 16]. The efficacy of guselkumab in patients with an inadequate response to ustekinumab and versus secukinumab has also been demonstrated [14, 15].

Studies supporting the regulatory approvals of guselkumab were conducted in controlled settings and in patient populations that were restricted according to study eligibility criteria, and therefore may not be representative of real‐world clinical practice [17]. Evidence on the effectiveness and safety of guselkumab in patients with moderate‐to‐severe plaque psoriasis treated in routine clinical practice would be informative for therapeutic decision making. This observational study aimed to evaluate the effectiveness and safety of guselkumab in Korean patients with moderate‐to‐severe plaque psoriasis in a real‐world clinical setting. This study was conducted to fulfill the requirement of a local pharmacovigilance plan after regulatory drug approval according to the risk management plan in South Korea.

## 2 Methods

### 2.1 Study Design and Patients

This prospective, non‐interventional, observational study was conducted at 44 clinical centers in South Korea. Patients who received guselkumab according to the product label in the course of routine clinical practice were eligible [18]. Guselkumab is indicated for adult patients with moderate‐to‐severe plaque psoriasis who are candidates for phototherapy or systemic therapy in Korea. Exclusion criteria were enrollment in an interventional clinical study, prior history of guselkumab treatment, and contraindication to guselkumab per the approved product label. Patient selection bias was mitigated by enrolling participants consecutively as they attended regular clinic visits.

Guselkumab was administered by subcutaneous injection as per the approved product label. The recommended dose is 100 mg at weeks 0 and 4, and every 8 weeks thereafter [9]. Patients were followed for up to 56 weeks (see Figure S1 for the study design). The primary data source was the patients' medical records; all data were entered into case report forms. The frequency and timing of patient visits were in accordance with routine clinical practice.

This study was conducted in accordance with the International Society for Pharmacoepidemiology Guidelines for Good Pharmacoepidemiology Practices. The protocol and any amendments, and the patient informed consent form, were approved by the local institutional review board/independent ethics committee at participating sites. All patients provided written informed consent before participation.

### 2.2 Outcomes

Demographic and disease characteristics (disease history, medical history, previous therapies) were recorded at visit 1 (baseline, week 0). Data for the following parameters were collected at visits 1 (baseline, week 0), 2 (approximately week 4), and 3–7 (approximately every 8 weeks after week 4, weeks 12–44): Guselkumab administration status; concomitant medications; effectiveness parameters (Psoriasis Areas and Severity Index [PASI], BSA affected, and Investigator's Global Assessment [IGA]); patient‐completed patient‐reported outcome (PRO) instruments (administered as available at each clinical practice; Dermatology Life Quality Index [DLQI] and Medication Satisfaction Questionnaire [MSQ]). Data for parameters, excluding guselkumab administration status, were also collected at treatment discontinuation. Safety was monitored from the first dose of guselkumab to 12 weeks after the last dose (i.e., week 56), or until loss to follow‐up, withdrawal of consent, or death, whichever occurred first.

Since this was a real‐world study, the frequency and timing of treatment administration and patient visits were in accordance with routine clinical practice, and as such, may not have occurred precisely every 4 or 8 weeks per label instructions. Treatment was generally administered within ±2 weeks of the label‐defined treatment intervals by healthcare professionals at hospitals; hence, the second injection was administered 4 ± 2 weeks (visit 2) after visit 1 and then 8 ± 2 weeks after each previous treatment (visits 3–7) over the duration of the present study [9, 10].

### 2.3 Statistical Analyses

Sample size was determined assuming an incidence rate of serious infection/malignancy of 0.6% [11]. Serious infection and malignancy are the important potential safety risks for guselkumab identified based on results from all clinical trials. The sample size was set at 600 patients, targeting 20% more than the 500 patients calculated to detect at least two cases of serious infection/malignancy with 80% statistical power to account for patients who discontinued participation over the course of the study.

Effectiveness was evaluated for all participants who received at least one dose of study drug and had both baseline and at least one post‐baseline effectiveness evaluation (effectiveness analysis set). Safety was evaluated for all participants who received at least one dose of study drug (safety analysis set).

Effectiveness data were summarized at baseline and at each visit using descriptive statistics and compared using paired *t*‐test or Wilcoxon signed‐rank test for PASI, BSA, and DLQI. The proportions of participants with IGA 0/1 and MSQ levels of satisfaction or dissatisfaction (extremely, very, somewhat, and neither satisfied nor dissatisfied) at baseline and each visit were summarized and compared using McNemar's test. Drug survival (i.e., the rate and duration of adherence to the study drug) was assessed using Kaplan–Meier statistics. Factors predicting PASI75, PASI90, or PASI100 responses at week 44 (visit 7) were assessed by univariable analysis; those found to be predictive at *p* < 0.1 were further evaluated by multivariable logistic regression analysis, with age, sex, and PASI score at baseline as covariates. Safety data were summarized as numbers and percentages of adverse events (AEs) occurring during the study period; incidence rates with 95% confidence intervals (CIs) were calculated. Analyses were two‐sided and *p* values < 0.05 were considered statistically significant. Statistical analyses were performed using statistical software package SAS 9.4 (SAS Institute, Cary, NC, USA).

## 3 Results

### 3.1 Patient Population

Between February 25, 2019 and March 25, 2022, 717 Korean patients with plaque psoriasis were screened, and 707 patients who met eligibility criteria were enrolled (Figure S2). At the data cutoff date (August 23, 2022), 562 (79.5%) patients had completed the 56‐week study observation period. Of the 145 (20.5%) patients who discontinued early, loss to follow‐up (*n* = 53, 36.6%), discontinuation of guselkumab (*n* = 45, 31.0%), and patient withdrawal of consent (*n* = 2, 1.4%) were the main reasons for leaving the study. The safety analysis set comprised 707 patients. The effectiveness analysis set comprised 531 patients; 176 patients were excluded because they had PASI < 10 or BSA < 10% (*n* = 132) at baseline or did not have both baseline and post‐baseline effectiveness data (*n* = 44) (Figure S2).

The overall population had a mean ± standard deviation (SD) age of 45.8 ± 13.9 years and was predominantly male (*n* = 477, 67.5%); mean ± SD disease duration was 119.9 ± 114.8 months (Table 1). The most common comorbidities were hypertension (*n* = 82, 11.3%) and rheumatic autoimmune disease (*n* = 75, 10.6%), and two (0.3%) patients had co‐existing palmoplantar pustulosis. The majority of patients had PASI ≥ 10 (570/674, 84.6%) and BSA ≥ 10% (572/659, 86.8%) at baseline. Most had received prior systemic therapy (*n* = 631, 89.3%); other common prior treatments were phototherapy (*n* = 494, 69.9%) and topical therapy (*n* = 427, 60.4%). The most common systemic oral agents previously used were cyclosporin A (*n* = 414, 58.6%) and methotrexate (*n* = 330, 46.7%). Prior biologic therapy had been received by 236/707 (33.4%) patients, and 471 (66.6%) had never been treated with a biologic agent (Table 1).

**TABLE 1:** Summary of patient baseline demographic and disease characteristics.

Category (unless stated otherwise, percentages are based on *N*)	Overall population *N* = 707	Biologic naïve *N* = 471	Biologic experienced *N* = 236	*p* ^a^
Age, years				
Mean ± SD	45.8 ± 13.9	44.4 ± 13.5	48.4 ± 14.3	0.0008
Sex, *n* (%)				
Male	477 (67.5)	313 (66.5)	164 (69.5)	0.4163
Female	230 (32.5)	158 (33.5)	72 (30.5)	
BMI				
*n* ^b^	366	219	147	0.5400
Mean ± SD	25.2 ± 3.7	25.2 ± 3.9	25.3 ± 3.5	
Disease duration, months				
Mean ± SD	119.9 ± 114.8	104.3 ± 105.0	151.1 ± 126.8	< 0.0001
Concurrent psoriatic arthropathy				
Yes	62 (8.8)	25 (5.3)	37 (15.7)	< 0.0001
No	645 (91.2)	446 (94.7)	199 (84.3)	< 0.0001
Comorbidity				
Diabetes mellitus	47 (5.6)	30 (6.4)	17 (7.2)	0.6747
Cardiovascular disease	11 (1.6)	8 (1.7)	3 (1.3)	0.7595
Impaired glucose regulation	4 (0.6)	1 (0.2)	3 (1.3)	0.0767
Hypertension	82 (11.3)	50 (10.6)	32 (13.6)	0.2490
Hyperlipidemia	59 (8.3)	38 (8.1)	21 (8.9)	0.7066
Autoimmune thyroiditis^b^	8 (1.1)	2 (0.4)	6 (2.5)	0.0192
Inflammatory bowel disease	1 (0.1)	0 (0)	1 (0.4)	0.3338
Rheumatic autoimmune disease	75 (10.6)	35 (7.4)	40 (16.9)	0.0001
Uveitis	1 (0.1)	1 (0.2)	0 (0)	1.0000
Malignancy	4 (0.6)	1 (0.2)	3 (1.3)	0.1109
Non‐alcoholic fatty liver disease	4 (0.6)	1 (0.2)	3 (1.3)	0.1109
Psychiatric disorders	15 (2.1)	5 (1.1)	10 (4.2)	0.0057
Palmoplantar pustulosis	2 (0.3)	2 (0.4)	0 (0)	0.5546
Baseline PASI score				
*n* ^b^	674	446	228	< 0.0001
Mean ± SD	14.0 ± 6.5	15.8 ± 6.2	10.6 ± 5.6	
Baseline BSA score				
*n* ^b^	658	438	220	< 0.0001
Mean ± SD	17.8 ± 13.1	20.7 ± 12.9	12.2 ± 11.5	
Baseline IGA				
*n* ^b^	385	247	138	< 0.0001
Moderate	168 (43.6)	121 (49.0)	47 (34.1)	
Severe	29 (7.5)	19 (7.7)	10 (7.2)	
Baseline DLQI				
*n* ^b^	408	270	138	< 0.0001
0–1	22 (5.4)	6 (2.2)	16 (11.6)	
2–5	61 (15.0)	29 (10.7)	32 (23.2)	
6–10	76 (18.6)	50 (18.5)	26 (18.8)	
11–20	153 (37.5)	110 (40.7)	43 (31.2)	
21–30	96 (23.5)	75 (27.8)	21 (15.2)	
Prior treatment history				
Systemic oral agent	631 (89.3)	435 (92.4)	196 (83.1)	0.0002
Phototherapy	494 (69.9)	330 (70.1)	164 (69.5)	0.8757
Topical	427 (60.4)	287 (60.9)	140 (59.3)	0.6794
Biologics	236 (33.4)	0 (0)	236 (100)	—
Prior lines of biologic therapy				
0	471 (66.6)	471 (100)	0 (0)	—
1	199 (28.1)	0 (0)	199 (84.3)	
2	30 (4.2)	0 (0)	30 (12.7)	
≥ 3	7 (1.0)	0 (0)	7 (3.0)	
Concomitant therapies				
Systemic oral agent	74 (10.5)	34 (7.2)	40 (16.9)	< 0.0001
Steroid	7 (1.0)	5 (1.1)	2 (0.8)	1.0000
Methotrexate	4 (0.6)	0 (0.0)	4 (1.7)	0.0122
Cyclosporin A	17 (2.4)	10 (2.1)	7 (3.0)	0.4902
Acitretin	1 (0.1)	0 (0.0)	1 (0.4)	0.3338
Other	56 (7.9)	25 (5.3)	31 (13.1)	0.0003
Phototherapy	8 (1.1)	4 (0.8)	4 (1.7)	0.4514
Topical therapy	337 (47.7)	195 (41.4)	142 (60.2)	< 0.0001

Abbreviations: BMI, body mass index; BSA, body surface area; DLQI, Dermatology Life Quality Index; IGA, Investigator's Global Assessment; PASI, Psoriasis Area and Severity Index; SD, standard deviation.

^a^ *p* values reflect differences between the biologic‐naïve and biologic‐experienced patients.

^b^ The number of available patients per each variable; this was the denominator for percentages for associated variables.

Demographic and baseline disease characteristics were generally comparable between the subgroups of patients with and without prior biologic therapy (Table 1), although some imbalances between the two groups were noted. Compared with the biologic‐naïve group, respectively, the biologic‐experienced group was older (mean ± SD 44.4 ± 13.5 vs. 48.4 ± 14.3 years; *p* = 0.0008) and had a longer disease duration (104.3 ± 105.0 vs. 151.1 ± 126.8 months; *p* < 0.0001), lower PASI score (15.8 ± 6.2 vs. 10.6 ± 5.6; *p* < 0.0001), and lower BSA (20.7% ± 12.9% vs. 12.2% ± 11.5%; *p* < 0.0001), and a greater proportion had received a systemic oral agent (*n* = 34 [7.2%] vs. *n* = 40 [16.9%]; *p* < 0.0001) or topical therapy (*n* = 195 [41.4%] vs. *n* = 142 [60.2%]; *p* < 0.0001). Most biologic‐experienced patients (*n* = 199, 84.3%) had received just one prior line of biologic therapy; seven (3.0%) had received ≥ 3 prior lines (Table 1).

### 3.2 Effectiveness Analyses

#### 3.2.1 PASI Responses

**FIGURE 1:**
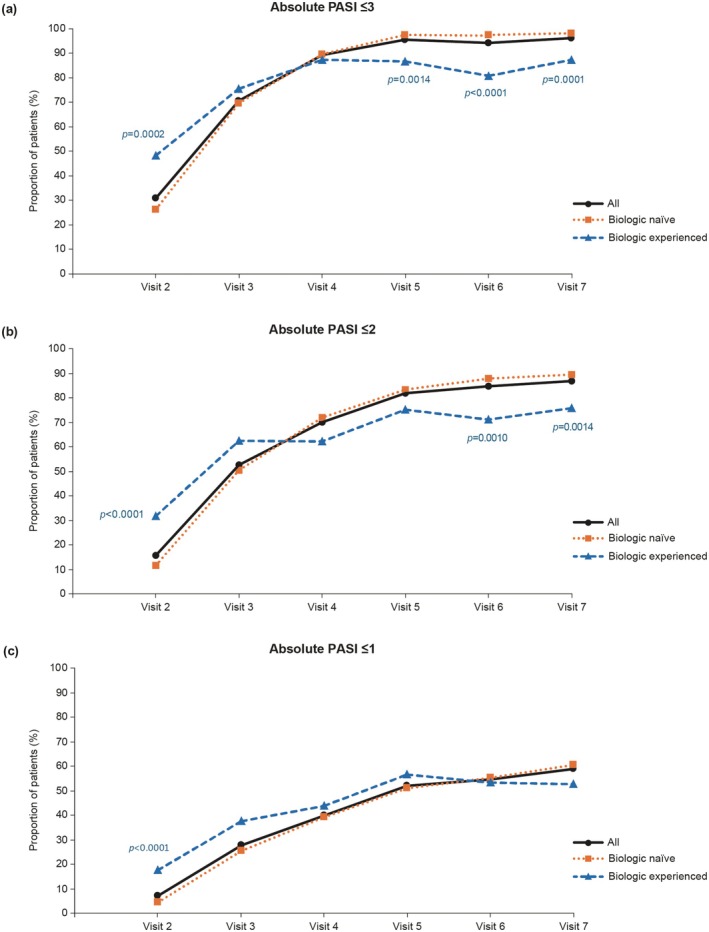
Proportions of patients with absolute PASI scores of ≤ 3 (a), ≤ 2 (b), and ≤ 1 (c) by visit in the overall population (effectiveness analysis set) and by prior biologic use. *p* values reflect differences between the biologic‐naïve and biologic‐experienced patients. PASI, Psoriasis Area and Severity Index.

The proportion of patients with an absolute PASI of ≤ 3, ≤ 2, or ≤ 1 increased over time, reaching a maximum of 96.0%, 86.7%, and 59.0%, respectively, at visit 7 in the overall population (Figure 1). A significantly greater proportion of biologic‐naïve (vs biologic‐experienced) patients achieved an absolute PASI of ≤ 3 by visits 5–7 (*p* = 0.001 at visit 7) and absolute PASI of ≤ 2 by visits 6–7 (*p* = 0.0014 at visit 7). The proportion of patients with a PASI75 response increased after the start of treatment, reaching 98.5% at visit 7. The proportions of patients with PASI90 and PASI100 responses also increased over time, reaching 74.3% and 25.4%, respectively, at visit 7 (Figure S3). Compared with the biologic‐experienced group, a significantly greater proportion of biologic‐naïve patients achieved PASI75 (biologic‐naïve vs. biologic‐experienced, 99.4% vs. 94.9%, *p* = 0.0141) and PASI90 (78.0% vs. 59.0%, *p* = 0.0006) responses at visit 7.

#### 3.2.2 IGA Response

**FIGURE 2:**
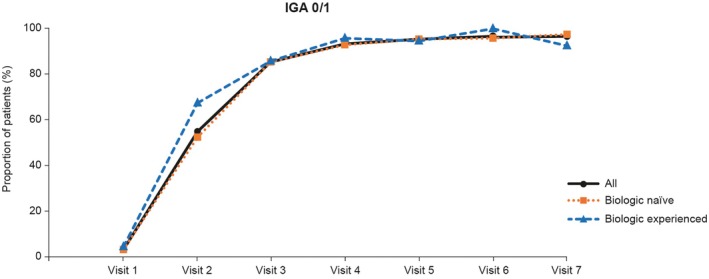
Proportions of patients achieving an IGA score of 0 or 1 by visit in the overall population (effectiveness analysis set) and by prior biologic use. IGA, Investigator's Global Assessment.

IGA responses (IGA 0 [cleared], IGA 1 [minimal], or IGA 2 [mild]) also improved over time with guselkumab treatment. By visit 5, all patients in the overall population had achieved an IGA score of 2 or lower. The proportion of patients who achieved IGA 0/1 increased over time, in both the biologic‐naïve and biologic‐experienced patient groups (Figure 2). IGA 0/1 was achieved by 96.5% (221/229) of all patients at visit 7, and by 97.4% (184/189) and 92.5% (37/40) of biologic‐naïve and biologic‐experienced patients, respectively, with no significant difference between these two subgroups.

#### 3.2.3 DLQI

DLQI score decreased over time in the overall population, reaching a mean score of 2.0 ± 3.1 (*n* = 234) at visit 7 (Table S1). The pattern of improvement over time was similar regardless of history of biologic therapy; the biologic‐naïve and biologic‐experienced groups achieved mean scores of 1.9 ± 3.1 (*n* = 188) and 2.4 ± 3.1 (*n* = 46), respectively, at visit 7. The proportion of patients achieving DLQI 0/1 increased after the start of treatment and was 64.1% (150/234 patients) at visit 7 for the overall population. Improvement in DLQI 0/1 response over time was seen in both biologic‐naïve and biologic‐experienced groups (Table S2). At visits 6 and 7, a greater proportion of biologic‐naïve patients achieved DLQI 0/1 compared with biologic‐experienced patients.

#### 3.2.4 Drug Survival

**FIGURE 3:**
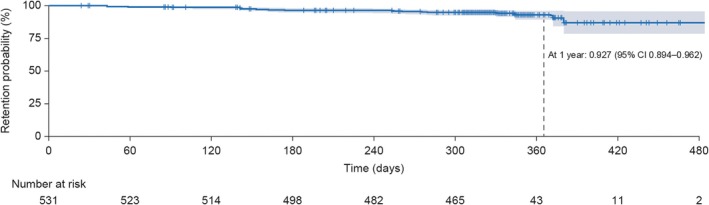
Estimates of guselkumab drug survival over time. CI, confidence interval.

Drug survival rates for guselkumab were high throughout the study observation period. The estimated drug survival rate at 1 year was 92.7% (Figure 3). The reasons for treatment discontinuation are presented in Table S3. The most common reasons for discontinuation were study completion and loss to follow‐up.

#### 3.2.5 Correlation Analysis

In the univariable logistic regression analysis, factors that may predict PASI90 response at visit 7, based on a significance threshold of *p* < 0.10, were sex; chest or nail psoriasis morphology; baseline PASI score; baseline IGA score indicating moderate or severe disease; a baseline MSQ score of 2; prior treatment with phototherapy, topical therapy, and biologic therapy; and concomitant treatment with a systemic oral agent or topical therapy (Table 2). The only factors that were still predictive of PASI90 response at visit 7 in the multivariable logistic regression analysis, at a significance threshold of *p* < 0.05, were sex (female vs. male, odds ratio [OR], 2.36; *p* = 0.0499), nail morphology (yes vs. no, OR, 0.37; *p* = 0.0237), baseline total PASI score (OR, 1.18; *p* = 0.0087), baseline IGA score indicating moderate or severe disease (moderate vs. minimal, OR, 3.58; *p* = 0.0009; severe vs. minimal, OR, 0.26, *p* = 0.0098 respectively), and concomitant treatment with a topical therapy (yes vs. no, OR, 0.23; *p* = 0.0004) (Table 2).

**TABLE 2:** Predictors of PASI90 response at visit 7 by univariable and/or multivariable analysis.^a^ Listed are variables with *p* < 0.1 in the univariable analysis, which were subsequently included in the multivariable logistic regression analysis.^b^

PASI90 variable,^c^ *N* = 405	Univariable analysis^a^			Multivariable analysis^b^		
	Odds ratio^a^	95% CI	*p*	Odds ratio^a^	95% CI	*p*
**Sex: Female (vs. male)**	**1.55**	**0.93–2.58**	**0.0929**	2.36	**1.00–5.56**	**0.0499**
Age (years)	0.99	0.97–1.00	0.1332	1.02	0.99–1.05	0.1387
Psoriasis morphology (vs. no)						
Chest	1.65	1.05–2.59	0.0307	2.00	0.88–4.56	0.0979
**Nail**	**0.54**	**0.31–0.93**	**0.0269**	**0.37**	**0.16–0.88**	**0.0237**
**Baseline PASI** **score—total**	**1.07**	**1.02–1.12**	**0.0093**	**1.18**	**1.04–1.33**	**0.0087**
Baseline IGA (vs. minimal)						
**Moderate**	**6.41**	**1.27–32.45**	**< 0.0001**	**3.58**	**0.57–22.50**	**0.0009**
**Severe**	**0.68**	**0.12–3.87**	**0.0287**	**0.26**	**0.03–2.07**	**0.0098**
Baseline MSQ = 2 (vs. MSQ = 1)^d^	0.51	0.13–2.06	0.0183	—	—	—
Prior treatment (vs. no)						
Phototherapy	1.71	1.07–2.73	0.0246	0.94	0.42–2.07	0.8711
Topical therapy	1.91	1.22–3.00	0.0049	1.35	0.60–3.04	0.4670
Biologic therapy	0.41	0.24–0.68	0.0007	1.29	0.52–3.22	0.5870
Concomitant treatment (vs. no)						
Systemic oral agent	0.47	0.22–0.99	0.0477	0.37	0.10–1.40	0.1444
**Topical** **therapy**	**0.22**	**0.14–0.36**	**< 0.0001**	**0.23**	**0.10–0.52**	**0.0004**

*Note:* The bold values signify variables that were predictive of PASI90 response by both uni‐ and multivariable analysis.

Abbreviations: CI, confidence interval; IGA, Investigator's Global Assessment; MSQ, Medication Satisfaction Questionnaire; PASI, Psoriasis Area and Severity Index; PASI90, 90% improvement in Psoriasis Area and Severity Index.

^a^ These analyses were conducted using logistic regression.

^b^ Variables with *p* < 0.1 on univariable analysis were included in multiple logistic regression analysis, with age, sex, and PASI score at baseline as covariates.

^c^ Categorical variables were modeled using effect coding, whereby each level represents deviations from the overall mean.

^d^ MSQ = 2 is “very dissatisfied”; MSQ = 1 is “extremely dissatisfied”.

Factors predictive of PASI100 or PASI75 response at visit 7, at the significance threshold of *p* < 0.10, in the univariable analysis are listed in Tables S4 and S5, respectively. The only factors still predictive of PASI100 response at visit 7 in multivariable analysis, at a significance threshold of *p* < 0.05, were nail psoriasis (*p* = 0.0052), prior phototherapy (*p* = 0.0062), and concomitant topical therapy (*p* = 0.0143) (Table S4). The only factor still predictive of PASI75 response in the multivariable analysis was prior biologic therapy (*p* = 0.0249) (Table S5).

### 3.3 Safety

Cumulative follow‐up over the course of the study was 581.8 patient years (PY), with a median follow‐up time of 0.9 years. There were 245 AEs reported in 159 (22.5%) patients, with an incidence rate (95% CI) of 42.1 (38.1–46.1) per 100 PY. The most frequent AEs were pruritus (23 patients, 4.0 [95% CI 2.4–5.5] per 100 PY), urticaria (10 patients, 1.7 [95% CI 0.7–2.8] per 100 PY), folliculitis (8 patients, 1.4 [95% CI 0.4–2.3] per 100 PY), and arthralgia (8 patients, 1.4 [95% CI 0.4–2.3] per 100 PY) (Table 3). Most AEs each occurred in just one patient. A total of 14 serious AEs (SAEs) were reported in 13 (1.8%) patients, with an incidence rate (95% CI) of 2.4 (1.2–3.7) per 100 PY. A full list of SAEs is provided in Table S6. The most frequent type of SAE (by System Organ Class) was gastrointestinal disorders, which occurred in three (0.4%) patients. By preferred term, no SAE occurred in more than one patient. Regarding AEs of special interest: Malignancies with onset after study start were reported for two (0.3%) patients, one with breast cancer and another with prostate cancer; a single cardiovascular AE, palpitations, was reported in one (0.1%) patient. There was one case of serious infection, reported as enterocolitis infectious (Table S6).

**TABLE 3:** Crude incidence rate of adverse events in more than two patients per 100 PY.

Adverse events (safety analysis set; *N* = 707)	Patients, *n* (%)		Events (*n*)	Incidence per 100 PY (%)	95% CI
Any	159	(22.5)	245	42.1	38.1–46.1
Pruritus	23	(3.3)	23	4.0	2.4–5.5
Urticaria	10	(1.4)	10	1.7	0.7–2.8
Arthralgia	8	(1.1)	8	1.4	0.4–2.3
Folliculitis	8	(1.1)	8	1.4	0.4–2.3
Drug ineffective	5	(0.7)	5	0.9	0.1–1.6
Headache	5	(0.7)	5	0.9	0.1–1.6
Tenia pedis	5	(0.7)	5	0.9	0.1–1.6
Herpes simplex	4	(0.6)	4	0.7	0.0–1.4
Psoriasis	4	(0.6)	4	0.7	0.0–1.4
Abdominal pain	3	(0.4)	3	0.5	0.0–1.1
Acne	3	(0.4)	3	0.5	0.0–1.1
Alopecia	3	(0.4)	3	0.5	0.0–1.1
Benign prostatic hyperplasia	3	(0.4)	3	0.5	0.0–1.1
Bony tinea	3	(0.4)	3	0.5	0.0–1.1
Dermatophytosis of the nail	3	(0.4)	3	0.5	0.0–1.1
Dizziness	3	(0.4)	4	0.7	0.0–1.4
Hand dermatitis	3	(0.4)	3	0.5	0.0–1.1
Herpes zoster	3	(0.4)	3	0.5	0.0–1.1
Nausea	3	(0.4)	3	0.5	0.0–1.1
Onychomycosis	3	(0.4)	3	0.5	0.0–1.1
Pain in extremity	3	(0.4)	3	0.5	0.0–1.1
Upper respiratory tract infection	3	(0.4)	3	0.5	0.0–1.1

Abbreviations: CI, confidence interval; PY, patient years.

## 4 Discussion

In this large, prospective, observational study conducted over a period of approximately 1 year, guselkumab demonstrated a high level of effectiveness and was well‐tolerated in Korean patients with moderate‐to‐severe plaque psoriasis treated in routine clinical practice. The findings from this study complement the evidence obtained from pivotal clinical trials for guselkumab in psoriasis. Guselkumab conferred durable responses, with 96.0%, 86.7%, and 59.0% of patients achieving absolute PASI scores of ≤ 3, ≤ 2, and ≤ 1, respectively, and 96.5% of patients having minimal or clear disease by visit 7. Absolute PASI is a frequently used endpoint for evaluating effectiveness of psoriasis treatments in clinical practice, and it has been suggested that absolute PASI ≤ 3 may be of particular clinical relevance [19]. Furthermore, guidelines for psoriasis treatment recommend targeting an absolute PASI goal as part of any treatment plan [20, 21, 22, 23, 24].

This study also demonstrated high proportions of patients achieving PASI75 (98.5%), PASI90 (74.3%), and IGA 0/1 (96.5%) responses at visit 7 (approximately week 44). In general, results were consistent with those obtained from randomized controlled clinical trials of patients with moderate–to‐severe psoriasis. Although patients in the present study had a shorter disease duration and less severe disease based on baseline BSA, and PASI and IGA scores compared with those in clinical trials, a consistent treatment effect was seen. In the global phase III VOYAGE 1 study, 87.8% and 76.3% of patients achieved PASI75 and PASI90 responses, respectively, and 80.5% achieved IGA 0/1 at week 48 with guselkumab—each higher than the respective response rates achieved with adalimumab [11]. In ECLIPSE, 92%, 84%, and 85% of patients receiving guselkumab achieved PASI75, PASI90, and IGA 0/1 at week 48, respectively [15]. NAVIGATE provided additional evidence for patients with an inadequate response to ustekinumab, showing that a higher proportion achieved a PASI90 response at week 28 after switching to guselkumab than those continuing with ustekinumab [14].

In this study, over half of the patients reported a DLQI 0/1 response at visit 4 (approximately week 20), with an increased response rate at visit 7 (approximately week 44), consistent with improvements in DLQI response reported in clinical trials [12, 14]. Achievement of low absolute PASI scores corresponded with improvements in PROs, including the DLQI, such that for most patients psoriasis had either no or relatively little effect on daily life after treatment with guselkumab. In this context, the results reported in this study indicate that treatment goals are being successfully attained with guselkumab treatment in routine real‐world practice in Korea.

The impact of race/ethnicity on the effectiveness of guselkumab remains equivocal. One recent systematic review found that the efficacy of biologics for treating moderate‐to‐severe plaque psoriasis varied according to race/ethnicity and the particular biologic. Regarding guselkumab, IGA 0/1 was achieved by a greater proportion of White patients, compared with Asian, Black, or Latino patients [25]. However, a subgroup analysis of the VOYAGE 1 and VOYAGE 2 studies concluded that responses to guselkumab were comparable between Asian and non‐Asian patients with psoriasis [26]. Furthermore, genetic and non‐genetic factors underlying the pathogenesis of the disease may differ between Asian and non‐Asian patients, which may contribute to reported differences in efficacy between races/ethnicities. Compared with patients with psoriasis from non‐Asian countries (Europe and the USA), Asian patients have a lower bodyweight and body mass index, and more severe psoriasis. In addition, there is a male predominance in Asian patients, whereas it is generally understood that the prevalence of psoriasis is equal among males and females elsewhere in the world [27, 28, 29, 30]. Comparison of the baseline characteristics in the present study versus those in some other real‐world studies from Europe [31, 32, 33] revealed that Korean patients had a shorter disease duration, lower BMI, different profile of comorbidities, and slightly different baseline disease severity. These characteristics were similar to those of another real‐world study of Korean patients [34]. Comparison of the findings of the present study with other real‐world studies of guselkumab may provide insights into racial differences in treatment response to guselkumab in the real‐world setting. In an observational, retrospective, single‐center cohort study in Korea that included biologic‐naïve patients, PASI90 and PASI100 responses at week 56 with guselkumab treatment were observed in 91.3% and 82.6% of patients, respectively [34]. An observational study in Spain that included predominantly biologic‐experienced patients reported PASI90 and PASI100 responses at week 24 in 59.4% and 49.0% of patients, respectively; the proportions of patients achieving an absolute PASI score of ≤ 4 and ≤ 2 at week 24 were 85.9% and 77.9%, respectively [31]. In the current study, it was observed that treatment response was sustained over a longer follow‐up period, indicating that the effectiveness of guselkumab remained consistent and durable over time. Similarly, a study in the Czech Republic including both biologic‐naïve and ‐experienced patients reported that 94.8% and 70.7% of patients had absolute PASI scores of ≤ 3 and ≤ 1, respectively, at 36 months [32]. In another study in Belgium, the proportions of PASI75, PASI90, and PASI100 responders at week 88 were 95.5%, 91%, and 73%, respectively [33]. Although between‐study comparisons should be made with caution, the previous and present studies indicate no apparent differences in clinical response to guselkumab in Asian and Western populations.

Conversely, the PASI100 response rate at visit 7 (equivalent to approximately week 44) with guselkumab treatment in our study (25.4%) was lower than those from other clinical trials and real‐world studies. In VOYAGE 1, 47.4% of patients achieved a PASI100 response at week 48 [11], and in VOYAGE 2, a PASI100 response was observed in 44.2% of patients at week 24 [12]. The difference in PASI100 response rates could be attributed to variations in baseline PASI score, study design, patient population, prior treatment, and real‐world practices regarding the use of biologics among countries.

Guselkumab was effective in both biologic‐naïve and biologic‐experienced patients, but trending toward better responses in patients without prior use of biologic therapy. There was a significant difference in the proportion of patients with an absolute PASI score of ≤ 3 between biologic‐naïve and biologic‐experienced patients at visit 7, which is notable as absolute PASI ≤ 3 may have particular clinical relevance [19]. Similarly, in a real‐world setting in Chinese patients, guselkumab also demonstrated effectiveness regardless of prior biologic use. However, no prior history of biologic use was significantly associated with achievement of PASI90, PASI100, and absolute PASI < 3 responses at week 20 [19]. Overall, response rates were high in the biologic‐naïve group, consistent with responses demonstrated in the pivotal clinical trials of guselkumab. Clinical responses in the present study were robust and generally consistent with the results from clinical trials for both biologic‐naïve and biologic‐experienced patients; in particular, the more substantial response in biologic‐naïve patients supports guselkumab as an effective first‐line biologic therapy for patients with psoriasis in the real‐world setting [11, 12, 14, 15, 26].

Factors predictive of PASI90 response in this study were female sex, nail psoriasis, baseline PASI total score, baseline IGA score indicating moderate or severe disease, and concomitant treatment with a topical therapy. Evidence of improved treatment outcomes in females compared with males has been reported previously in a large patient sample (*n* = 5346) [35], although this remains controversial [36]. Factors that could underly a sex difference in response to systemic therapies include differences in disease status, hormonal status, immune response, comorbidities, and treatment adherence [37, 38]. Nail psoriasis is a difficult‐to‐treat condition that is associated with prolonged and severe psoriasis, severe pain, and decreased quality of life, and may be a risk factor for psoriatic arthritis [39]. It is unclear whether the frequent use of topical therapies to treat nail psoriasis [40] may explain the reduced response (i.e., likelihood of achieving a PASI90 response) to guselkumab. The present findings suggest that to a point, the higher the baseline disease severity, the greater the probability of achieving a PASI90 response (a trend that was also seen in patients with a moderate baseline IGA score). However, in patients with very severe disease, the probability of achieving a PASI90 response was reduced. This may be due to the higher probability of previous biologic use, disease duration, and presence of comorbidities. Similar findings have been reported in a pooled analysis of clinical trial findings for patients with moderate‐to‐severe plaque psoriasis treated with apremilast, whereby patients with moderate disease were more likely to achieve a PASI score of ≤ 2 and had higher rates of PASI response compared with their counterparts with more severe skin disease [41]. That said, guselkumab remains sufficiently effective even in patients with severe disease. Since it may be challenging to demonstrate sufficient efficacy (i.e., PASI90 response) with increasing disease severity, active treatment may be necessary in these patients. Baseline PASI and IGA scores in patients with moderate or severe psoriasis have been identified as predictors of response to guselkumab in a study of super‐responders, with odds ratios (95% CI) of 0.97 (0.955–0.993; *p* = 0.007) and 0.66 (0.433–0.997; *p* = 0.048), respectively [42]. The correlation between baseline disease severity and response to guselkumab remains uncertain. In the real‐world setting, concomitant use of topical therapies is not uncommon (e.g., 47.7% of patients in the present study) and is likely due to inadequate response to a systemic therapy [6, 43]. This may explain the potentially negative impact on the ability to achieve a PASI90 response with guselkumab. These patients may have more severe or resistant disease, and thus possibly a reduced likelihood of achieving a PASI90 response with guselkumab. Further study into factors predicting response is needed, toward establishing patient prognosis.

The present study demonstrates positive findings with guselkumab over a relatively short, 1‐year follow‐up; longer‐term follow‐up is needed to provide robust data on the efficacy and safety of continued guselkumab treatment for this chronic disease. A long‐term analysis of the global VOYAGE 1 and VOYAGE 2 trials (*n* = 1829 patients with moderate‐to‐severe psoriasis) demonstrated that the efficacy with guselkumab observed at week 48 of treatment was sustained through 5 years [16]. The proportion of patients achieving a PASI90 response in the two studies was 84.1% and 82.0%, respectively, and 82.4% and 85.0%, respectively, achieved IGA 0 or 1. Findings were similar for patients who crossed over from adalimumab to receive guselkumab. Health‐related quality of life benefits during the double‐blind periods of both studies were also maintained over the longer term. The rate of guselkumab retention was high, with > 80% of those who received at least one dose having continued treatment for 5 years [16]. Consistent with this is the finding in the present study of an estimated guselkumab survival of 92.7% at 1 year. A pooled safety analysis of VOYAGE 1 and VOYAGE 2 for an additional 5 years of follow‐up (up to week 264) revealed a consistently favorable safety profile with no new safety signals; rates of AEs were low, including for those of particular interest in psoriasis (serious infections, nonmelanoma skin cancer, other malignancies, major adverse cardiovascular events) [44]. These long‐term efficacy and safety findings were confirmed in a *post hoc* analysis of Asian patients in VOYAGE 1 and VOYAGE 2 [13]. Long‐term data reported in a real‐world clinical setting were consistent with the findings for these clinical trials. A retrospective assessment of guselkumab in patients in clinical practice (Italy) with moderate‐to‐severe psoriasis demonstrated improvements in disease that were maintained up to week 144 [45].

In the present study, the drug survival rate for guselkumab was found to be remarkably high, with a 92.7% survival rate observed at 1 year. In comparison, a real‐world observational study in the Netherlands found that the overall 1‐ and 2‐year drug survival rates for adults with plaque psoriasis treated with guselkumab were 85.5% and 77.8%, respectively. When discontinuation due to lack of effectiveness was considered, the drug survival rates for guselkumab were 92.8% at 1 year and 88.7% at 2 years; and when discontinuation due to AEs was considered, the drug survival rates for guselkumab were 94.3% at 1 year and 92.1% at 2 years [46]. The drug survival rates reported herein may represent an underestimation of the true rates as a result of adopting the conservative approach of classifying cases with unclear reasons for discontinuation as events.

Treatment with guselkumab was well‐tolerated. The incidence rates of AEs and the most common AEs reported were in line with other real‐world guselkumab studies [19, 31, 32, 34] and pivotal trials [11, 12, 14, 15]. There were no major cardiovascular events, and the incidence of malignancies was within the range previously reported in other studies (0.2%–0.6%) [11, 12, 14, 15]. In contrast to the pivotal trials for guselkumab, no cases of non‐melanoma skin cancer were documented in this study. The safety findings of the present study are consistent with those of several pooled analyses of global trials and retrospective database studies, including with long‐term follow‐up, that have consistently demonstrated no significant safety concerns with guselkumab [13, 31, 32, 44, 45, 47, 48]. Some of these studies reported rates (per 100 PY) of 148–346 for AEs overall (vs. 42.1 in the present study), 0.7–1.6 for treatment discontinuation, and 3.1–5.3 for SAEs (vs. 2.4 in the present study). Rates of AEs with guselkumab were low and stable over time (up to 5 years of follow‐up), and similar to those reported for placebo. Taken together, these findings highlight the good tolerability of guselkumab in this patient population.

Strengths of this study include a large sample size, a multicenter design that enhances generalizability, and a comprehensive analysis of disease status, including both patients who have and have not previously received biologic therapy. In addition, it is particularly noteworthy that this study focuses on real‐world data and extends over a relatively long‐term period of follow‐up through 1 year of treatment with guselkumab, especially when considering its relevance to Korean patients. However, several limitations should also be considered. The absence of a control group impacts interpretation of effectiveness outcomes, and missing data may affect the overall accuracy and completeness of the findings. In addition, the study did not analyze psoriasis responses based on anatomical regions affected or the types of prior biologics received, which might limit the understanding of treatment effectiveness in specific patient groups. Lastly, as may be the case in any real‐world study, under‐reporting of less common AEs may occur.

## 5 Conclusions

Guselkumab administered under approved label recommendations was effective and well‐tolerated in Korean patients with moderate‐to‐severe plaque psoriasis in a real‐world clinical setting, regardless of prior biologic therapy experience, with no new safety signals identified.

## Ethics Statement

The protocol and any amendments, and the patient informed consent form, were approved by the local institutional review board/independent ethics committee at participating sites.

## Consent

All patients provided written informed consent before participation.

## Conflicts of Interest

J.A. and Y.K. are employees of Janssen Korea Ltd. The other authors declare no conflicts of interest.

## Supporting information


Appendix S1.
**Table S1.** Change in the DLQI score over time (effectiveness analysis set).
**Table S2.** Proportion of patients achieving a Dermatology Life Quality Index score of 0 or 1 over time (effectiveness analysis set).
**Table S3.** Treatment discontinuation.
**Table S4.** Predictors of PASI100 response at visit 7 by univariable and/or multivariable analysis^a^. Listed are variables with *p* < 0.1 in univariable analysis, which were subsequently included in multiple logistic regression^b^.
**Table S5.** Predictors of PASI75 response at visit 7 by univariable and/or multivariable analysis^a^. Listed are variables with *p* < 0.1 in univariable analysis, which were subsequently included in multiple logistic regression^b^.
**Table S6.** Crude incidence rate of serious adverse events per 100 patient years.
**Figure S1.** Study design.
**Figure S2.** Study disposition.

## Data Availability

The data from this study are not publicly available and no data sharing is planned in order to protect the privacy of study participants.
