# Supplementary material for: Effectiveness and Safety of Guselkumab in Patients With Moderate‐to‐Severe Plaque Psoriasis in Real‐World Practice in Korea: A Prospective, Multicenter, Observational, Postmarketing Surveillance Study
Source: J Dermatol. 2025 Apr 28;52(7):1125–37. doi: 10.1111/1346-8138.17757 (PMC12231943; doi:10.1111/1346-8138.17757)
Supplement: Supplementary file 1 — Appendix S1. Table S1. Change in the DLQI score over time (effectiveness analysis set). Table S2. Proportion of patients achieving a Dermatology Life Quality Index score of 0 or 1 over time (effectiveness analysis set). Table S3. Treatment discontinuation. Table S4. Predictors of PASI100 response at visit 7 by univariable and/or multivariable analysisa. Listed are variables with p < 0.1 in univariable analysis, which were subsequently included in multiple logistic regressionb. Table S5. Predictors of PASI75 response at visit 7 by univariable and/or multivariable analysisa. Listed are variables with p < 0.1 in univariable analysis, which were subsequently included in multiple logistic regressionb. Table S6. Crude incidence rate of serious adverse events per 100 patient years. Figure S1. Study design. Figure S2. Study disposition. [file JDE-52-1125-s001.docx]

SUPPLEMENTARY MATERIAL

Effectiveness and safety of guselkumab in patients with moderate-to-severe plaque psoriasis in real-world practice in Korea: a prospective, multicenter, observational, postmarketing surveillance study

Bong Seok Shin, Miri Kim, Moo Kyu Suh, Young Bok Lee, Sang Woong Youn, Ji Yeoun Lee, Chul Woo Kim, Ga Young Lee, Sang Wook Son, Kwang Ho Kim, Jihye An, Youngdoe Kim, Kwang Joong Kim, Dong Hyun Kim

**Table of contents**

[Supplementary Table S1. Change in the DLQI score over time (effectiveness analysis set) 3](#_Toc180600168)

[Supplementary Table S2. Proportion of patients achieving a Dermatology Life Quality Index score of 0 or 1 over time (effectiveness analysis set) 4](#_Toc180600169)

[Supplementary Table S3. Treatment discontinuation 5](#_Toc180600170)

[Supplementary Table S4. Predictors of PASI100 response at visit 7 by univariable and/or multivariable analysis^a^. Listed are variables with p<0.1 in univariable analysis, which were subsequently included in multiple logistic regression^b^. 6](#_Toc180600171)

[Supplementary Table S5. Predictors of PASI75 response at visit 7 by univariable and/or multivariable analysis^a^. Listed are variables with *p*<0.1 in univariable analysis, which were subsequently included in multiple logistic regression^b^. 8](#_Toc180600172)

[Supplementary Table S6. Crude incidence rate of serious adverse events per 100 patient years. 9](#_Toc180600173)

[Supplementary Figure S1. Study design 10](#_Toc180600174)

[Supplementary Figure S2. Study disposition 11](#_Toc180600175)

[Supplementary Figure S3. Proportion of patients achieving 75%, 90%, or 100% improvement in Psoriasis Area and Severity Index (PASI) by visit (effectiveness analysis set) in all patients 12](#_Toc180600176)

## Supplementary Table S1. Change in the DLQI score over time (effectiveness analysis set)

| Measurement timepoint^a^ | Statistics | Overall population N=531 | Biologic-naïve N=415 | Biologic-experienced N=116 | *p*^b^ |
| --- | --- | --- | --- | --- | --- |
| Visit 1 | n | 313 | 254 | 59 | 0.0349 |
|  | Mean (SD) | 14.7 (8.1) | 15.2 (8.0) | 12.6 (8.5) |  |
| Visit 2 | n | 269 | 226 | 43 | 0.4944 |
|  | Mean (SD) | 8.9 (6.8) | 9.1 (6.8) | 8.2 (6.7) |  |
| Visit 3 | n | 254 | 214 | 40 | 0.2816 |
|  | Mean (SD) | 4.6 (5.6) | 4.7 (5.8) | 3.7 (4.4) |  |
| Visit 4 | n | 287 | 233 | 54 | 0.5872 |
|  | Mean (SD) | 3.0 (4.5) | 2.9 (4.5) | 3.2 (4.4) |  |
| Visit 5 | n | 226 | 190 | 36 | 0.3749 |
|  | Mean (SD) | 2.3 (3.7) | 2.3 (3.8) | 2.4 (3.1) |  |
| Visit 6 | n | 204 | 167 | 37 | 0.0323 |
|  | Mean (SD) | 2.1 (3.6) | 2.0 (3.8) | 2.6 (3.0) |  |
| Visit 7 | n | 234 | 188 | 46 | 0.0726 |
|  | Mean (SD) | 2.0 (3.1) | 1.9 (3.1) | 2.4 (3.1) |  |

^a^Visit 1 was baseline (week 0); visit 2 was 4 ±2 weeks after visit 1; visits 3–7 were 8±2 weeks after each previous visit.

^b^*p* values reflect differences between the biologic-naïve and biologic-experienced patients.

DLQI, Dermatology Life Quality Index; SD, standard deviation.

## Supplementary Table S2. Proportion of patients achieving a DLQI score of 0 or 1 over time (effectiveness analysis set)

| Measurement timepoint^a^  Data are n/n’ (%) | Overall population N=531 | | Biologic-naïve N=415 | | Biologic-experienced N=116 | | *p*^b^ |
| --- | --- | --- | --- | --- | --- | --- | --- |
| Visit 1 | 12/313 | (3.8) | 6/254 | (2.4) | 6/59 | (10.2) | 0.0128 |
| Visit 2 | 35/269 | (13.0) | 26/226 | (11.5) | 9/43 | (20.9) | 0.0922 |
| Visit 3 | 100/254 | (39.4) | 81/214 | (37.9) | 19/40 | (47.5) | 0.2516 |
| Visit 4 | 163/287 | (56.8) | 133/233 | (57.1) | 30/54 | (55.6) | 0.8384 |
| Visit 5 | 141/226 | (62.4) | 120/190 | (63.2) | 21/36 | (58.3) | 0.5837 |
| Visit 6 | 134/204 | (65.7) | 116/167 | (69.5) | 18/37 | (48.6) | 0.0158 |
| Visit 7 | 150/234 | (64.1) | 126/188 | (67.0) | 24/46 | (52.2) | 0.0599 |

^a^Visit 1 was baseline (week 0); visit 2 was 4 ±2 weeks after visit 1; visits 3–7 were 8±2 weeks after each previous visit.

^b^*p* values reflect differences between the biologic-naïve and biologic-experienced patients.

DLQI, Dermatology Life Quality Index; n/n’, number of patients achieving response/number of patients available per timepoint.

## Supplementary Table S3. Treatment discontinuation

| Category | N |
| --- | --- |
| Total censored | 500 |
| Pregnancy | 1 |
| Treatment termination with symptom improvement | 2 |
| Withdrawal of consent | 1 |
| Follow up loss | 65 |
| Study completion | 431 |
| Total events censored | 31 |
| Adverse event | 3 |
| Discontinuation of guselkumab^a^ | 28 |
| Total | 531 |

^a^Discontinuation of Tremfya treatment” was conservatively considered an event as the reason was not confired

## Supplementary Table S4. Predictors of PASI100 response at visit 7 by univariable and/or multivariable analysis^a^. Listed are variables with p<0.1 in univariable analysis, which were subsequently included in multiple logistic regression^b^.

|  | Univariable analysis^a^ | | | Multivariable analysis^b^ | | |
| --- | --- | --- | --- | --- | --- | --- |
| Variable^c^  N=405 | **Odds ratio^a^** | **95% CI** | ***p*** | **Odds ratio^a^** | **95% CI** | ***p*** |
| Sex: female (vs male) | 1.09 | 0.68–1.77 | 0.7170 | 0.88 | 0.51–1.52 | 0.6354 |
| Age, years | 1.00 | 0.98–1.02 | 0.8292 | 0.99 | 0.97–1.01 | 0.2867 |
| Psoriasis family history (vs no) | 1.80 | 0.91–3.57 | 0.0919 | 2.08 | 0.96–4.51 | 0.0644 |
| Psoriasis morphology (vs no) |  |  |  |  |  |  |
| Scalp | 0.60 | 0.38–0.94 | 0.0248 | 0.84 | 0.48–1.47 | 0.5424 |
| Face | 0.56 | 0.34–1.90 | 0.0157 | 0.69 | 0.36–1.32 | 0.2635 |
| Neck | 0.65 | 0.40–1.05 | 0.0810 | 1.17 | 0.62–2.23 | 0.6296 |
| Chest | 0.56 | 0.36–0.89 | 0.0129 | 0.64 | 0.36–1.16 | 0.1442 |
| Lower arm | 0.63 | 0.38–1.05 | 0.0735 | 1.11 | 0.60–2.06 | 0.7416 |
| Palm | 0.35 | 0.14–0.93 | 0.0344 | 0.77 | 0.26–2.30 | 0.6377 |
| Back of hand | 0.45 | 0.25–0.82 | 0.0094 | 0.64 | 0.31–1.30 | 0.2162 |
| Nail | **0.22** | **0.09–0.53** | **0.0007** | **0.25** | **0.10–0.66** | **0.0052** |
| Buttock | 0.64 | 0.41–1.02 | 0.0577 | 1.23 | 0.70–2.19 | 0.4714 |
| Concurrent psoriatic arthropathy (vs no) | 0.33 | 0.16–0.69 | 0.0033 | 0.87 | 0.11–6.63 | 0.8891 |
| Comorbid rheumatic autoimmune disease (vs no) | 3.15 | 1.58–6.28 | 0.0011 | 5.24 | 0.80–34.50 | 0.0849 |
| Baseline PASI score – Total | 0.94 | 0.90–1.00 | 0.0153 | 0.95 | 0.90–1.00 | 0.0604 |
| Baseline BSA score – Total | 0.98 | 0.96–1.00 | 0.0204 | – | – | – |
| Baseline DLQI score – Total | 1.04 | 1.00–1.08 | 0.0459 | – | – | – |
| Baseline IGA = severe (vs minimal) | 0.07 | 0.01–0.91 | 0.0492 | – | – | – |
| Baseline MSQ = 2 (vs MSQ 1) ^d^ | 0.19 | 0.07–0.99 | 0.0582 | – | – | – |
| Prior treatment (vs no) |  |  |  |  |  |  |
| Phototherapy | **2.02** | **1.18–3.45** | **0.0100** | **2.28** | **1.26–4.11** | **0.0062** |
| Topical therapy | 1.93 | 1.19–3.14 | 0.0081 | 1.48 | 0.85–2.57 | 0.1635 |
| Concomitant topical treatment (vs no) | **0.55** | **0.35–0.88** | **0.0119** | **0.51** | **0.29–0.87** | **0.0143** |

^a^These analyses were conducted using logistic regression

^b^Variables with *p*<0.1 on univariable analysis were included in multiple logistic regression analysis, with age, sex and PASI score at baseline as covariates.

^c^Categorical variables were modeled using effect coding, whereby each level represents deviations from the overall mean.

^d^MSQ=2 is “very dissatisfied”; MSQ=1 is “extremely dissatisfied.”

BSA, body surface area affected; CI, confidence interval; DLQI, Dermatology Life Quality Index; IGA; Investigator’s Global Assessment; MSQ, Medication Satisfaction Questionnaire; PASI, Psoriasis Area and Severity Index; PASI100, 100% improvement in Psoriasis Area and Severity Index.

## Supplementary Table S5. Predictors of PASI75 response at visit 7 by univariable and/or multivariable analysis^a^. Listed are variables with *p*<0.1 in univariable analysis, which were subsequently included in multiple logistic regression^b^.

|  | Univariable analysis^a^ | | | Multivariable analysis^b^ | | |
| --- | --- | --- | --- | --- | --- | --- |
| PASI75 Variable^c^ | **Odds ratio^a^** | **95% CI** | ***p*** | **Odds ratio^a^** | **95% CI** | ***p*** |
| Sex: female (vs male) | 0.88 | 0.16-4.87 | 0.8844 | 0.76 | 0.13-4.65 | 0.7702 |
| Age, years | 1.03 | 0.96-1.10 | 0.4229 | 1.05 | 0.98-1.12 | 0.2149 |
| Concurrent psoriatic arthropathy (vs no)^d^ | 0.16 | 0.03–0.89 | 0.0367 | 0.17 | 0.03–1.06 | 0.0572 |
| Comorbid rheumatic autoimmune disease (vs no) | 0.19 | 0.03–1.09 | 0.0622 | – | – | NC^c^ |
| Baseline PASI score – Total | 1.00 | 0.87–1.15 | 0.9823 | 0.99 | 0.87–1.12 | 0.8317 |
| Prior biologic therapy (vs no) | **0.11** | **0.02–0.63** | **0.0131** | **0.13** | **0.02–0.77** | **0.0249** |
| Concomitant systemic therapy (vs no) | 0.16 | 0.03–0.92 | 0.0405 | 0.17 | 0.03–1.10 | 0.0625 |

^a^These analyses were conducted using logistic regression.

^b^Variables with *p*<0.1 on univariable analysis were included in multiple logistic regression analysis, with age, sex and PASI score at baseline as covariates.

^c^Categorical variables were modeled using effect coding, whereby each level represents deviations from the overall mean.

^d^In situations where the rate of achieving PASI75 is high and the rate of not achieving the event is very low, considering both concurrent psoriatic arthropathy and concurrent rheumatic autoimmune disease simultaneously can lead to multicollinearity issues, which in turn can cause problems in estimating the odds ratio (Spearman correlation = 0.91); therefore, only concurrent psoriatic arthropathy was included in the multivariable analysis and concurrent rheumatic autoimmune disease was not included.

CI, confidence interval; NC, not calculable; PASI, Psoriasis Area and Severity Index; PASI75, 75% improvement in Psoriasis Area and Severity Inde

## Supplementary Table S6. Crude incidence rate of serious adverse events per 100 PY.

|  | Overall population (N=707) | | |
| --- | --- | --- | --- |
| Serious adverse event  Preferred term | **No. of patients (%)** | **Events, n** | **Incidence rate per 100 PY (95% CI)** |
| Any | 13 (1.8) | 14 | 2.4 (1.2–3.7) |
| Anal hemorrhage | 1 (0.1) | 1 | 0.2 (0.0–0.51) |
| Chronic gastritis | 1 (0.1) | 1 | 0.2 (0.0–0.51) |
| Inguinal hernia | 1 (0.1) | 1 | 0.2 (0.0–0.51) |
| Pancreatitis acute | 1 (0.1) | 1 | 0.2 (0.0–0.51) |
| Breast cancer | 1 (0.1) | 1 | 0.2 (0.0–0.51) |
| Gastric adenoma | 1 (0.1) | 1 | 0.2 (0.0–0.51) |
| Hepatitis acute | 1 (0.1) | 1 | 0.2 (0.0–0.51) |
| Enterocolitis infectious | 1 (0.1) | 1 | 0.2 (0.0–0.51) |
| Meniscus cyst | 1 (0.1) | 1 | 0.2 (0.0–0.51) |
| Biopsy | 1 (0.1) | 1 | 0.2 (0.0–0.51) |
| Diabetes mellitus | 1 (0.1) | 1 | 0.2 (0.0–0.51) |
| Schizophrenia | 1 (0.1) | 1 | 0.2 (0.0–0.51) |
| Acute kidney injury | 1 (0.1) | 1 | 0.2 (0.0–0.51) |
| Ovarian cyst | 1 (0.1) | 1 | 0.2 (0.0–0.51) |

^a^Including cysts and polyps

CI, confidence interval; PY, patient year.

## Supplementary Figure S1. Study design


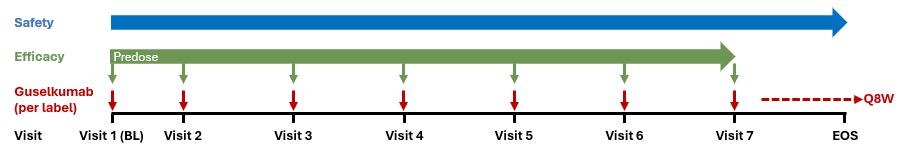


BL, baseline; EOS, end of study; Q8W, every 8 weeks (maintenance therapy).

## Supplementary Figure S2. Study disposition

**
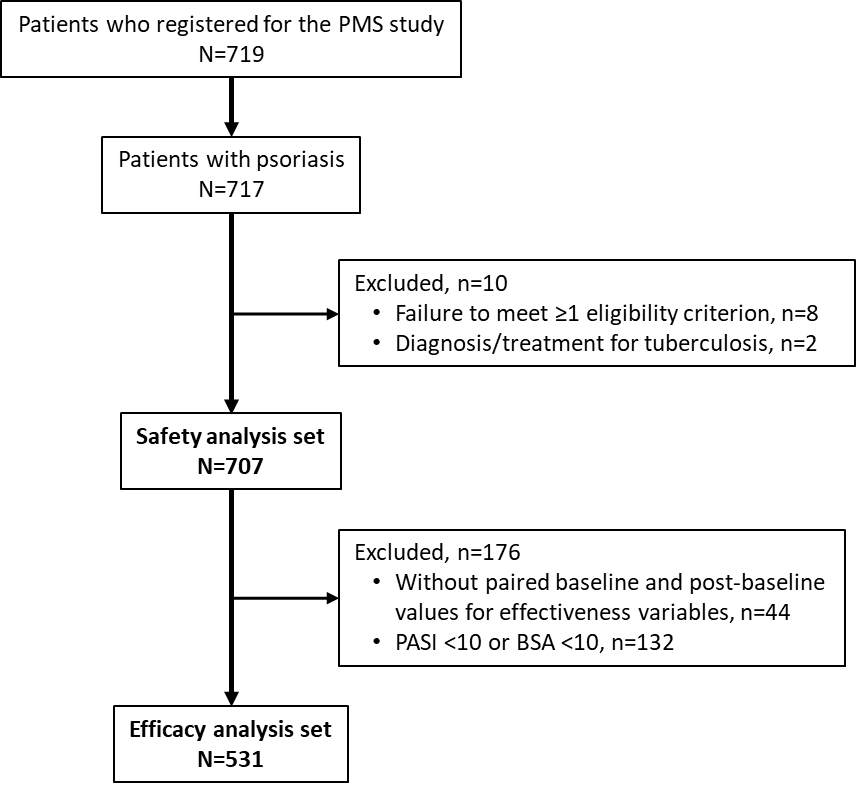
**

## BSA, body surface area; PASI, Psoriasis Area and Severity Index; PMS, postmarketing surveillance. Supplementary Figure S3. Proportion of patients achieving 75%, 90%, or 100% improvement in Psoriasis Area and Severity Index (PASI) by visit (effectiveness analysis set) in all patients


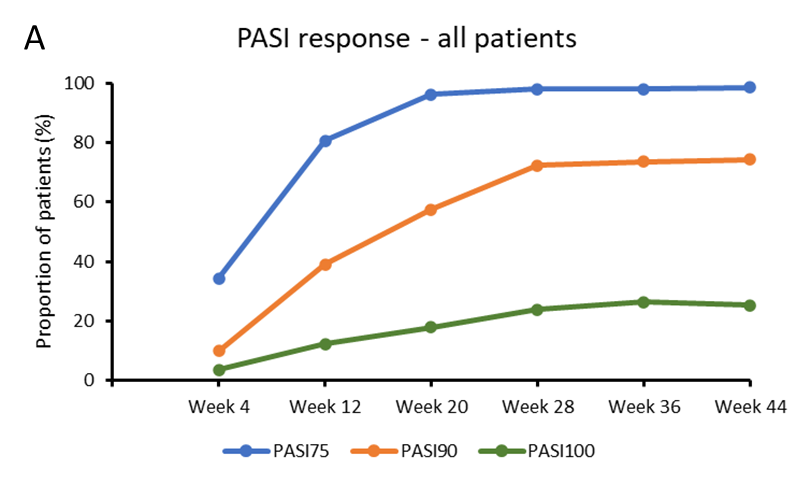


^a^*P* values reflect differences between the biologic-naïve and biologic-experienced patients.

PASI(75,90,100), (75%, 90%, 100%) improvement from baseline in Psoriasis Area and Severity Index.
